# Identification of Biomarker and Co-Regulatory Motifs in Lung Adenocarcinoma Based on Differential Interactions

**DOI:** 10.1371/journal.pone.0139165

**Published:** 2015-09-24

**Authors:** Ning Zhao, Yongjing Liu, Zhiqiang Chang, Kening Li, Rui Zhang, Yuanshuai Zhou, Fujun Qiu, Xiaole Han, Yan Xu

**Affiliations:** College of Bioinformatics Science and Technology, Harbin Medical University, Harbin, 150081, China; University of Erlangen-Nuremberg, GERMANY

## Abstract

Changes in intermolecular interactions (differential interactions) may influence the progression of cancer. Specific genes and their regulatory networks may be more closely associated with cancer when taking their transcriptional and post-transcriptional levels and dynamic and static interactions into account simultaneously. In this paper, a differential interaction analysis was performed to detect lung adenocarcinoma-related genes. Furthermore, a miRNA-TF (transcription factor) synergistic regulation network was constructed to identify three kinds of co-regulated motifs, namely, triplet, crosstalk and joint. Not only were the known cancer-related miRNAs and TFs (let-7, miR-15a, miR-17, TP53, ETS1, and so on) were detected in the motifs, but also the miR-15, let-7 and miR-17 families showed a tendency to regulate the triplet, crosstalk and joint motifs, respectively. Moreover, several biological functions (i.e., cell cycle, signaling pathways and hemopoiesis) associated with the three motifs were found to be frequently targeted by the drugs for lung adenocarcinoma. Specifically, the two 4-node motifs (crosstalk and joint) based on co-expression and interaction had a closer relationship to lung adenocarcinoma, and so further research was performed on them. A 10-gene biomarker (*UBC*, *SRC*, *SP1*, *MYC*, *STAT3*, *JUN*, *NR3C1*, *RB1*, *GRB2 and MAPK1*) was selected from the joint motif, and a survival analysis indicated its significant association with survival. Among the ten genes, JUN, NR3C1 and GRB2 are our newly detected candidate lung adenocarcinoma-related genes. The genes, regulators and regulatory motifs detected in this work will provide potential drug targets and new strategies for individual therapy.

## Introduction

Lung adenocarcinoma is a malignant cancer with the highest incidences and the worst prognosis. Several recent studies have used microarrays for genome-wide analyses of lung adenocarcinomas [1, 2], however these studies used methods based on the differential expression of genes, while ignoring changes in the molecular relationships among nonmalignant, early and advanced stages of disease [3], i.e., the so-called differential interactions.

In many cases, the occurrence of complex diseases is caused by multiple genes rather than a single one. Typically, the molecular regulations and interactions can vary according to tissue types and the different stages of disease development. Also, differences in molecular interactions between disease and control samples are not confined to a static level: in other words, changes in the intermolecular interactions may also be the cause of occurrence and/or development of diseases. Molecules binding to alternative partners in the molecular interaction network may be associated with disease. The study of differential interactions between molecules can detect important genes that may not be apparent under static conditions [4], therefore, it may not be appropriate to only separately consider the expression of each gene in diseased and normal states. In the identification of disease-related genes, the differential interactions between genes in the disease process should also be considered [5].

Transcription factors (TFs) play important roles in the regulation of gene expression. By binding to a specific region of the DNA sequence, TFs control the transcriptional activity of target genes. Prior studies of gene regulatory networks focused on the regulation of gene expression at the transcriptional level; however, increasing evidence has indicated that miRNAs also regulate gene expression at the post-transcriptional level [6]. Therefore, building a gene regulatory network that involves both transcriptional and post-transcriptional regulation is crucial. Prior studies on the synergistic regulation of miRNAs and TFs found a variety of significant motifs. involved in both processes, and all of these studies pointed out that these motifs serve as cornerstones in gene regulatory networks [7]. The protein interactome should also be considered in order to identify how the motifs affect downstream biological processes in gene regulatory networks. Thus, we should study the relationship between protein-protein interactions and their upstream regulators to deepen our understanding of biological metabolism.

McDoniels-Silvers et al. [8] found 92 differentially expressed genes (DEGs) in lung cancer by cDNA library screening and RNA analysis. Although, their experiments were highly accurate, they were very difficult and time consuming. Zhang et al. [2]detected 1,429 lung adenocarcinoma DEGs by bioinformatics methods. Liu et al. [5] improved the static method by considering differential expression and applied differential interaction analysis in disease research. From the dynamic perspective, they identified network modules or module biomarkers that included a set of genes related to gastric cancer. These three studies all achieved great results, but they did not consider transcriptional and post-transcriptional regulation of gene expression. Our previous study [9] established miRNA and TF co-regulatory networks, as well as identifying important regulators and significant miRNA-TF synergistic regulatory motifs. We found that the miR-17 family had an important effect on the proliferation and cell cycle regulation of non-small cell lung cancer. However, we did not consider the differential interactions.

The present study takes the differential interactions and miRNA-TF synergistic regulation of genes into account, and more comprehensively considers the regulation between biological molecules. Through this approach, not only were we able to verify the previous studies, but we were also able to detect the genes and regulators more closely linked with the occurrence and development of cancer. First, differential interaction genes (DIGs) were detected. We then predicted the miRNA/TF target genes in order to construct a miRNA-TF synergistic regulatory network. Depending on the regulatory relationships between molecules, three kinds of motifs (triplet, crosstalk and joint) were mined. Then, the topological properties and biological functions of the motifs were analyzed to find their similarities and differences. Finally, biomarkers and regulators related to lung adenocarcinoma were identified.

## Results

### The differential interactions can be used to detect new lung adenocarcinoma-related genes

In the present work, differential interaction between genes was considered in the disease process were considered during the identification of cancer-related genes. Three expression profiles were used to detect DIGs by applying a differential interaction analysis (**see** Materials and methods). The cancer-related genes detected by different microarray studies are often highly inconsistent [10, 11], even when there is not much technical noise and there is a wide differential expression in the cancer [12]. Therefore, the results of the three lung adenocarcinoma expression profiles were aggregated to obtain a comprehensive result. A total of 1,791 DIGs (S1 Table) were ultimately identified.

Most of the DIGs were associated with lung cancer, as confirmed by experiments or based on relevant literature. For instance, Bates et al. [13] demonstrated that BACA1 was lung cancer related, Jiang et al. [14] confirmed that TPM2 was a tumor suppressor gene, and Chen et al. [15] demonstrated that STAT1, ERBB3, and LCK were associated with lung cancer. The results confirmed the reliability of the detection of lung adenocarcinoma-related genes by differential interaction.

**Fig 1:**
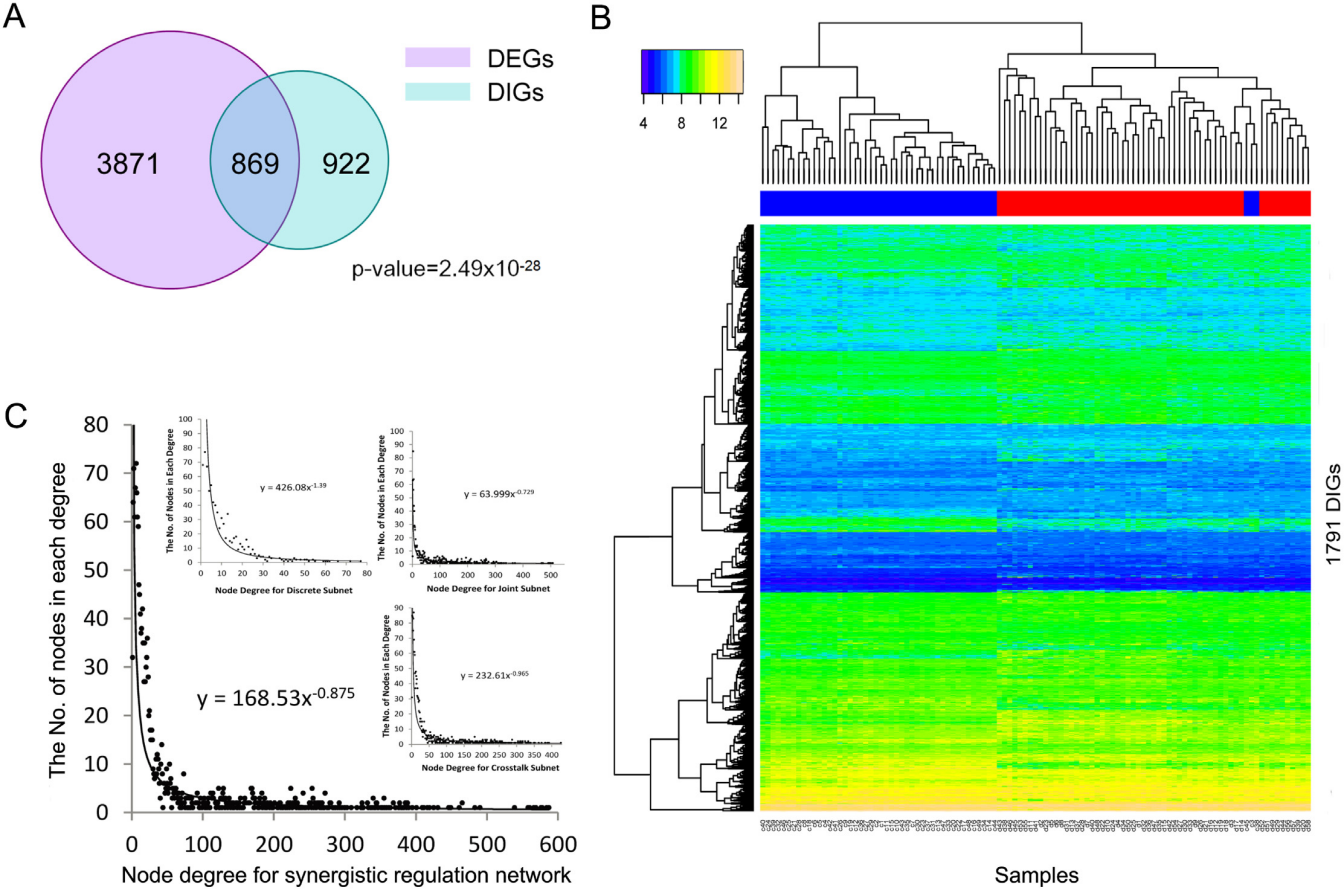
DIGs and the synergistic regulatory network are reliable. (A) The Venn diagram of DIGs and DEGs. The red circle represents DEGs and the blue circle represents DIGs. The amount of DEGs is quite smaller than DIGs. The overlap of them is significant. (B) The heatmap of samples (GSE10072) hierarchical clustering by 1,791 DIGs. The bar on the top of the heatmap indicates the group the samples really below to. Red represents disease and blue represents control. The sample orders under the heatmap corresponding to the orders in S5 Table. The 1,791 DIGs separate disease and control groups well. (C) Degree distributions of the synergistic regulatory network and each subnet. The large diagram indicates the degree distribution of synergistic regulatory network. Three insets from top to bottom, from left to right represent degree distributions of three subnets, triplet, crosstalk, and joint, respectively. They all met the requirement of scale-free network.

In order to further verify the accuracy of our results, we identified DEGs for the three profiles using the SAM [16] method (SAMR package) and took them as a combined unit. As shown in Fig 1A, the number of DEGs (4,686) was far greater than the number of DIGs (1,791), although a significant overlap (hypergeometric test p-value = 2.49 x 10^-28^) was noted between them. We also applied SAM and CoXpress [17] methods to GSE31547. They were chosen as they are mature methods representing differential expression and differential co-expression, respectively. We obtained 34 lung cancer-related genes from COSMIC as reference. A total of 834 genes were identified by our method, containing 11 COSMIC genes, while the results for SAM and CoXpress are only 1/1241 and 7/2216, respectively (S6 Table).

These indicated that, differential interaction could narrow the scope of experimental verification, and might be able to be used to detect new cancer-related genes that are missed by differential expression and differential co-expression studies.

To confirm the strong relationship between the 1,791 DIGs and lung adenocarcinoma, a hierarchical clustering was carried out (Fig 1B and S1 Fig). The results show that the disease samples and control samples could be separated more significantly with the 1,791 DIGs.

### The synergistic regulatory network and three kinds of motifs

In order to explore the transcriptional and post-transcriptional regulation of DIGs, a lung adenocarcinoma miRNA-TF synergistic regulatory network was constructed by combining the predicted target data of TFs and miRNAs with the relationships between DIGs. There were 305 miRNAs, 1,209 genes, 283 TFs, and 54,770 pairs of relationships in the network. As shown in Fig 1C, the fitting function of the degree distribution of the network followed the power-law distribution. This meets the standards of a scale-free network and suggests the biological property of this network. Therefore, this was considered an appropriate network for biological research.

In the present paper, three types of co-regulated motifs were defined based on the various relationships in the network: triplet, crosstalk, and joint. As defined in the Material and Methods section, triplet is a typical three-node feed-forward loop (FFL) with a single gene co-regulated by a miRNA and TF pair. Crosstalk and joint are both four-node motifs involving the co-expression and interaction of genes. The crosstalk and joint motifs are the two different modes of synergistic regulation of a miRNA and TF pair to two genes.

To test the significance of our motifs, 1,000 permutations were analyzed for the network. The motifs were mined from each random network, and the P-value and Z-value were calculated for each kind of the three motifs (**see** Materials and methods). The results showed that the P-values for each of the three motifs were below 0.001, and the Z-values were no less than 10. These results suggest that the excavated motifs are not likely to have arisen by chance.

To further understand the features and key regulators, motifs under each type were combined in three corresponding subnets. Corresponding to the motif types, the subnets were named triplet, crosstalk, and joint. The subnets contained 333, 1,156, and 618 genes, respectively. As shown in Fig 1C, the degree distribution of each subnet met the characteristics of scale-free network. Therefore, the hub nodes could play major roles in the network, which we investigated to further understand the subnets.

### Different miRNA families are involved in the regulation of the three different motifs

In the present work, hub nodes are defined as the nodes with the highest in-degree, out-degree and interactions in the network. The top ten hub miRNAs were detected for each subnet (Table 1). The miR-17 family (miR-17, miR-20a, miR-20b, miR-93, miR-106a, and miR-106b) were key regulators in all three subnets, especially in joint, and played important roles in lung adenocarcinoma. Four members of the miR-15 family (miR-15a, miR-15b, miR-16, and miR-195) were crucial regulators in triplet, and for Crosstalk, two let-7 family members (let-7a and let7b) had high degree values for crosstalk and regulated this subnet specifically. Thus the three subnets tended to be regulated by three different miRNA families.

**Table 1:** The hub miRNAs of each subnet.

Triplet	Crosstalk	Joint
hsa-miR-15a-5p	hsa-miR-497-5p	hsa-miR-20a-5p
hsa-miR-449a	hsa-miR-30b-5p	hsa-miR-106a-5p
hsa-miR-19a-3p	hsa-miR-195-5p	hsa-miR-17-5p
hsa-miR-15b-5p	hsa-miR-30c-5p	hsa-miR-106b-5p
hsa-miR-497-5p	hsa-let-7b-5p	hsa-miR-93-5p
hsa-miR-195-5p	hsa-miR-34a-5p	hsa-miR-130a-3p
hsa-miR-16-5p	hsa-let-7a-5p	hsa-miR-130b-3p
hsa-miR-18a-5p	hsa-miR-424-5p	hsa-miR-20b-5p
hsa-miR-19b-3p	hsa-miR-107	hsa-miR-301b
hsa-miR-182-5p	hsa-miR-20b-5p	hsa-miR-30e-5p

Note: The order of the miRNAs was based on degrees. The larger the degree was, the higher the rank was.

Nevertheless, although we had already chosen the miRNA-mRNA interactions supported by at least five experiments and shown to exist in at least three cancers to decrease the effect of false positives, the possibility that the hubs were caused by chance still could not be avoided. Therefore, we conducted a randomization test and a hypergeometric cumulative distribution test to ensure the biological significance of these hubs. The results showed that all of the hubs listed in Table 1 were significant in miRNA-mRNA interactions and are not hubs in random networks. This meant that the hubs were caused by biological significance rather than by false positives of the miRNA-target data. Similarly, the top ten hub TFs were extracted for each subnet (Table 2).

**Table 2:** The hub TFs of each subnet.

Triplet	Crosstalk	Joint
HIF1A	c-Myc	c-Myc
c-Myc	TP53	TP53
CREB1	SP1	SP1
ETS1	E2F-4	E2F-4
JUN	MYB	ETS1
RB1	REST	NFKB1
RBL2	TFAP2A	MYB
SP3	STAT1	E2F-1
STAT3	ETS1	JUN
TFAP2A	NFKB1	TFAP2A

Note: The order of the TFs was based on degrees. The larger the degree was, the higher the rank was.

### The co-regulation of hub miRNAs and TFs

The synergistic regulations between the hub regulators were studied. The miR-15, let-7, and miR-17 families were separately studied for triplet, crosstalk, and joint, respectively. The results showed that all of the three families co-regulated with MYC. The synergistic regulation of these miRNA families and MYC further confirmed their correlation with lung cancer. Also, four members of the E2F TF family (E2F1, E2F2, E2F3, and E2F4) were key regulators in the three subnets, confirming the synergistic regulation of the miR-17 family and the E2F transcription factor family. In crosstalk and joint, the two subnets involving co-expression and interaction, MYC, SP1, and TP53 had the most co-regulations with the corresponding miRNA family (the let-7 family for crosstalk and the miR-17 family for joint). Also, joint had some additional TFs compared to crosstalk, e.g., ETS1, MYB, STAT1, and so on, to co-regulate with its hub miRNA family.

### 4-node motifs are more closely associated with cancer

In the network, in order to understand how miRNAs and TFs participated in the various synergistic regulations, the top pairs of miRNA and TF were investigated. We assumed that the top 1% of miRNA and TF pairs that regulate the largest amount of related genes represent the characteristics of the subnet. Finally, from the three subnets (triplet, crosstalk, and joint) we obtained 134, 295, and 168 pairs of miRNA and TF, which were called the "cachets". The results are shown in the S2 Table.

Pathway and Gene Ontology (GO) annotation analyses were performed on the cachets of each subnet. After filtration, we paid attention to all the Kyoto Encyclopedia of Genes and Genomes (KEGG) pathways and the top 25% of GO annotations (S3 Table). The results exhibited that each subnet was annotated with cancer-related pathways and functions, but there were still differences between the subnets.

All of the three subnets were enriched in "regulation of the cell cycle" and the "response to stimulus". The two co-expression and interaction subnets, crosstalk and joint, were additionally enriched in the regulation of death, apoptosis, metabolic, transcription, cell proliferation, phosphorylation, biosynthetic processes, gene expression, and hemopoiesis. However, there still existed some differences between crosstalk and joint. For instance, joint was enriched in homeostasis and leukocyte differentiation, while crosstalk was enriched in macromolecular complex assembly and the ubiquitin-dependent protein catabolic process, the same as its pathway annotation.

From the qualitative viewpoint, all of the three subnets were all annotated with the "pathway in cancer" and "cell cycle". The two co-expression and interaction subnets, crosstalk and joint, were additionally annotated with MAPK, ErbB, p53, the T cell receptor, the B cell receptor and the chemokine signaling pathways, the adherens junction, and many kinds of cancers. Because the screening requirements of crosstalk were less strictly than joint, so that crosstalk had more extensive functions. In addition, crosstalk was annotated with Wnt, VEGF, TGF-beta and other signaling pathways, as well as leukocyte transendothelial migration, and natural killer cell mediated cytotoxicity. In particular, it was annotated with ubiquitin mediated proteolysis.

**Fig 2:**
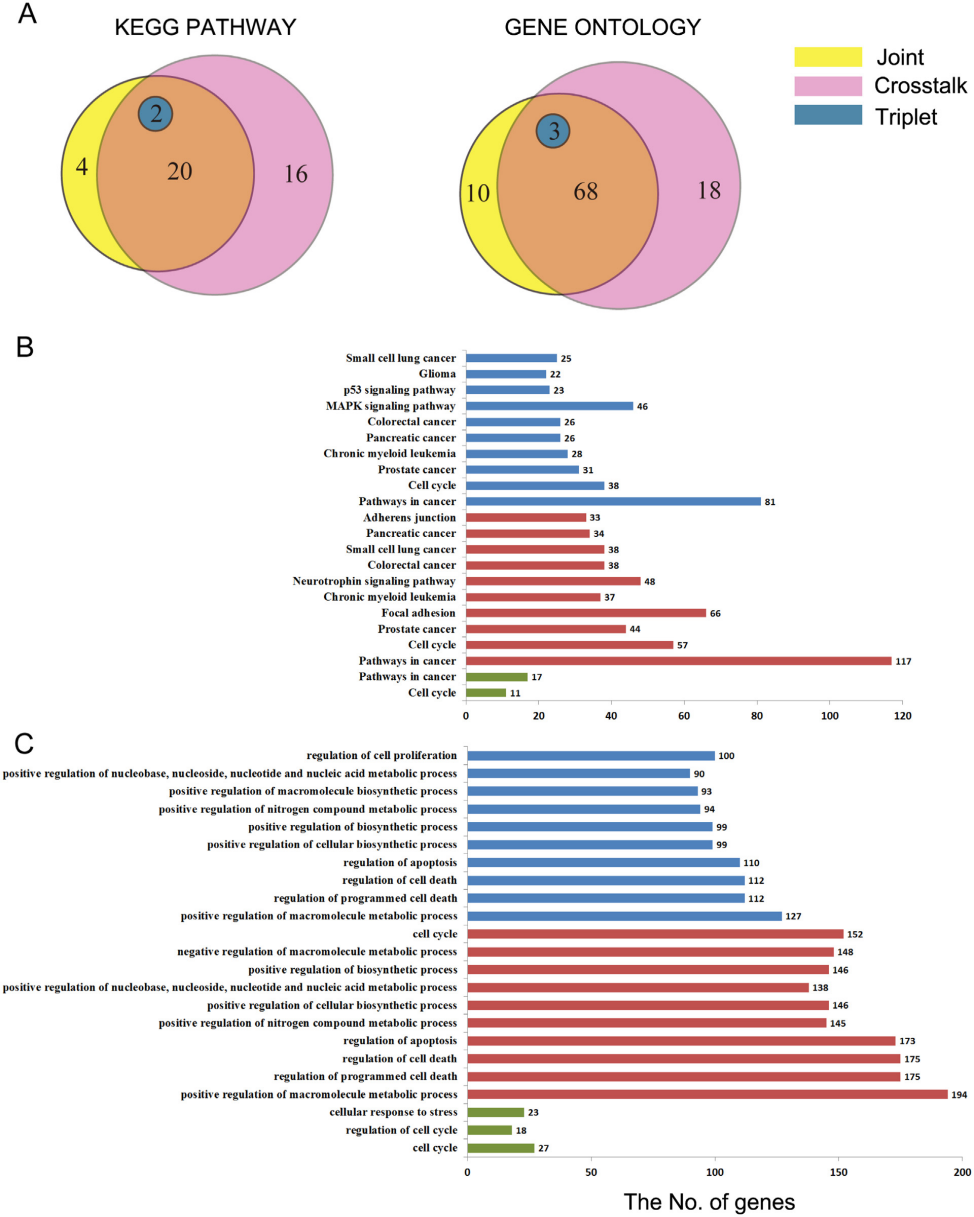
The KEGG&GO annotations of the three motifs. The Venn diagram indicates the number of pathways and GO terms that the three motifs annotated to. (B,C) The barplot of the total genes of the top ten terms that the three motifs annotated to. Green represents triplet, red represents crosstalk and blue represents joint. For the whole annotations, see S3 Table.

From the quantitative viewpoint, the functions of the co-expression and interaction subnets were more extensive and these two subnets had several functions in common (Fig 2). There were 11, 38, and 57 genes annotated to the cell cycle pathway for triplet, crosstalk, and joint, respectively. There were 26 and 18 genes annotated to the NSCLC pathway for crosstalk and joint, while triplet did not have any genes annotated to NSCLC (S3 Table). All the results suggested that we needed to pay more attention to crosstalk and joint.

### The 10-gene biomarker was associated with prognosis

The two co-expression and interaction subnets, crosstalk and joint, had functions more associated with cancer. As the internal intermolecular structure of joint was more closely regulated, so that joint was chosen for further study. Its ten hub genes were extracted (Table 3) and confirmed in the literature; seven of them (*UBC*, *SRC*, *SP1*, *MYC*, *STAT3*, *RB1*, and *MAPK1*) were verified to be associated with lung cancer, while the remaining three genes, *JUN*, *NR3C1*, and *GRB2*, were potentially new candidate lung adenocarcinoma-related genes. As lung adenocarcinoma is a complex disease that is affected by multiple genes, the ten genes were taken as a whole for use as a 10-gene biomarker. Second, KEGG and GO enrichment analysis was performed on the biomarker (S4 Table), and showed that it was enriched in functions significantly associated with cancer.

**Table 3:** The top ten genes of Joint.

Entrez ID	Gene Symbol
7316	UBC [54]
6714	SRC [55]
6667	SP1 [56]
4609	MYC [57]
6774	STAT3 [58]
3725	JUN
2908	NR3C1
5925	RB1 [59]
2885	GRB2
5594	MAPK1 [60]

Note: The order of the genes was based on degrees. The articles supported the association of genes to lung cancer were signed behind the gene symbols.

**Fig 3:**
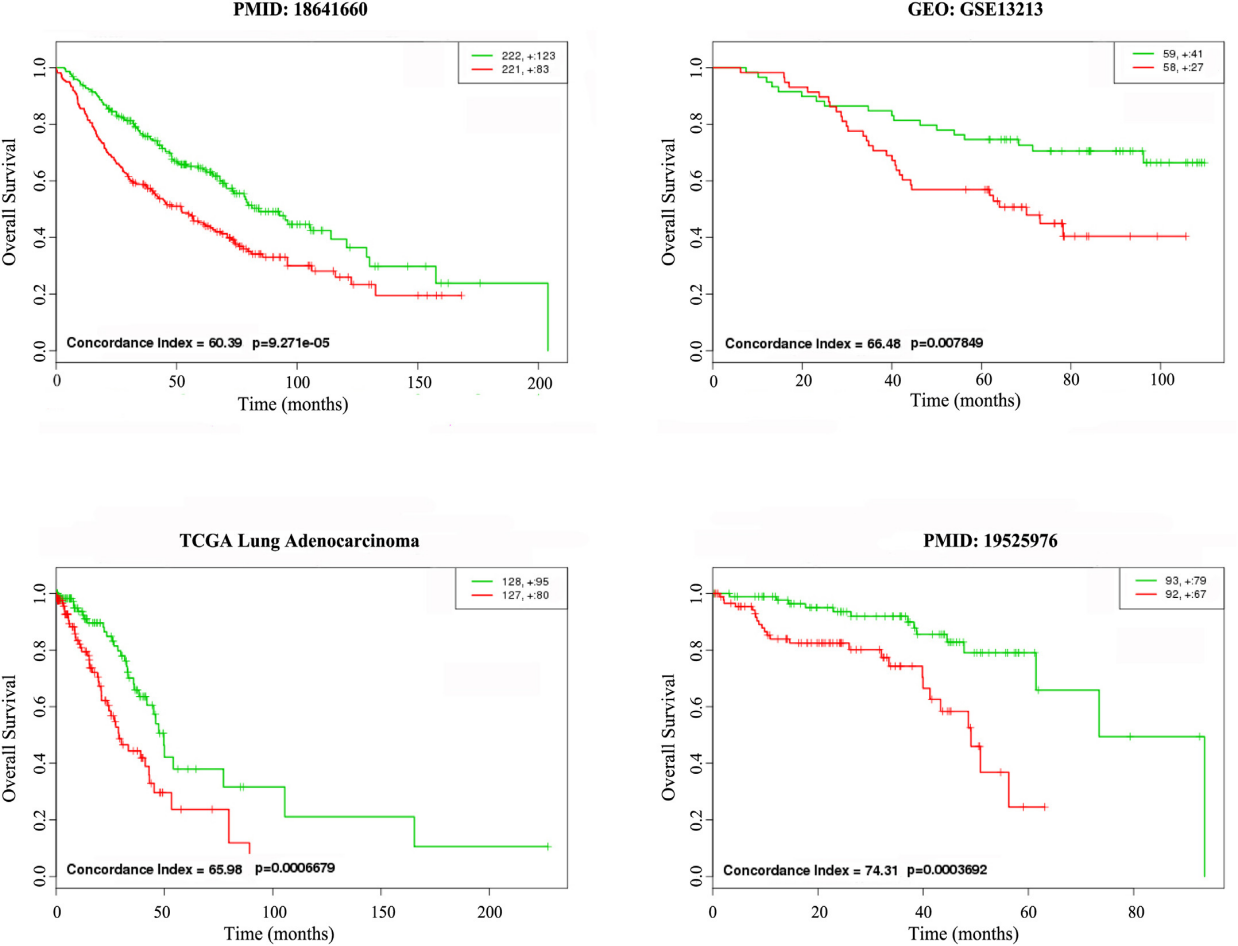
The survival analysis of ten hub genes of Joint. The “+” stands for the censoring samples. The X axis and Y axis respectively stands for observation time (months) and percent of survival people. Red and Green curves are high-risk group and low-risk group. The sources of data sets are on the top of each graph. Concordance Index (CI) and p-value are in the bottom-left insets.

Finally, to estimate the effect on prognosis of the 10-gene biomarker, survival analysis was performed to evaluate the potential for their correlation to lung adenocarcinoma. We selected four data sets for the survival analysis from the TCGA and GEO databases and from two literature sources (Fig 3). The results showed that the biomarker could easily distinguish the high-risk and low-risk groups in each of the four data sets. All of the p-values were significant (p-value = 9.27x10^-5^ for PMID: 18641660, p-value = 7.85x10^-3^ for GEO: GSE13213, p-value = 6.68x10^-4^ for TCGA lung adenocarcinoma, and p-value = 3.69x10^-4^ for PMID: 19525976). This suggested that the biomarker was tightly associated with lung adenocarcinoma.

As a comparison, the survival analysis of the top ten hub genes (Table 4) of crosstalk was also carried out for the four data sets. As expected, the prognostic ability of these ten genes was weaker than our 10-gene biomarker. Indeed, they could not even significantly distinguish the high and low risk groups of the TCGA samples (p-value = 0.02507) (S2 Fig).

**Table 4:** The top ten genes of Crosstalk.

Entrez ID	Gene Symbol
7534	YWHAZ
1026	CDKN1A
2033	EP300
3065	HDAC1
4609	MYC
2099	ESR1
595	CCND1
2885	GRB2
2908	NR3C1
5594	MAPK1

Note: The order of the genes was based on degrees.

In addition, in order to ensure 10 genes was an appropriate amount, the survival analysis was also carried out for the top five genes (S3 Fig) and the top twenty genes (S4 Fig), respectively. Neither could not distinguish the high-risk or low-risk groups in the data from an article (PMID: 18641660). The top five genes even have a non-significant p-value (p = 0.1123) for the data from GEO (GSE13213).

## Discussion

The regulations and interactions between molecules usually change according to the different tissues and stages of cancer. The changes in the intermolecular interactions may also be the cause of cancer development. Therefore, differential interactions were introduced to identify the lung adenocarcinoma-related genes. Studying the differences in the intermolecular interactions may allow the detection of important genes that cannot be detected under static conditions.

In the present work, the lung adenocarcinoma-related genes were detected by studying their differential interactions. The results showed that differential interactions could be used to reliably detect lung adenocarcinoma-related gene set containing significant genes that the use of differential expression could not detect. For example, *BRAF*, *ITGA3*, *PARK2*, *PIK3CA*, *RB1*, and *TGFB1* are the DIGs we detected. These are known lung cancer-related genes, but none of them were in the set of DEGs. Considering the difference between the disease and control samples at the dynamic level could be a supplement to analysis at the static level. Bandyopadhyay et al. [4] demonstrated that differential interaction could detect many gene functions that could not be detected under static conditions. Nicoloso et al. [18] found SNPs that could regulate gene expression through differential interactions. These findings confirm the feasibility of the detection of cancer-related genes by studying the differential interactions.

In this work, the co-expression and interaction between DIGs were considered to construct more comprehensive 4-node motifs. Sun et al. [19] demonstrated that the 4-node motifs were complementary to the 3-node motifs, and had a wider application in cancer research. Compared with 3-node FFLs, they found that the main impact of 4-node FFLs was the recruitment of more glioblastoma (GBM)-related genes and regulatory relationships into the regulatory network. This is consistent with the conclusions of our work. They also found that, 4-node FFLs tended to regulate genes belonging to the same biological processes. Similarly, the present study found that the function of the two 4-node motifs that considered co-expression and interaction, crosstalk and joint, were more closely associated with cancer.

Research into the hub regulators in the subnets of the three motifs showed that, the miR-15, let-7, and miR-17 families played important roles in triplet, crosstalk and joint, respectively. All of the three families have been reported to be associated with lung cancer, confirming the accuracy of our selection of hub genes, but previous research did not distinguish between the different modes of their regulation. Their co-regulation with MYC further confirmed their correlation with lung cancer. Two family members of miR-15 (miR-15a and miR-16) have been reported to be frequently downregulated in non-small cell lung cancer (NSCLC) and to affect cell cycle regulation [20], and are likely to regulate genes with TFs like triplet. However, let-7 and miR-17 are oncomiRs. The expressions of the oncogene *RAS* and let-7 show a reciprocal pattern, namely low let-7 and high *RAS* in cancerous cells, and high let-7 and low *RAS* in normal cells [21]. Reduced expression of let-7 family members is common in non-small cell lung cancer (NSCLC) [22, 23]. In the present work, we further found that they were likely to have a collaborative regulation with TFs like crosstalk. A high expression level of miR-17 family members induces cell proliferation, and the miR-17-92 cluster of the miR-17 family has repeatedly been reported overexpressed in NSCLC [24], whereas deletion of the miR-17-92 cluster in mice causes lethal lung and lymphoid cell developmental defects [25]. Our previous work also verified the correlation between the miR-17 family and NSCLC. This family preferred to coordinatedly regulate with TFs like joint. Therefore, miR-15 should participate less in the regulation of cancer than miR-17 and let-7. In addition, we confirmed the co-regulation of the miR-17 family and E2F TF family [26], which are involved in the cell cycle together with their co-regulated genes [27], where E2F and P53 can affect cell decisions [28]. The miR-17 family therefore offers the possibility to inhibit division and proliferation before restriction points.

The top ten hub TFs were extracted for each subnet (Table 2). The shared TFs were MYC, ETS1, and TFAP2A, with, MYC [29] and ETS1 [30] being reported to be lung cancer-related TFs. Although TFAP2A has not been explicitly reported to be associated with lung cancer, it has been associated with the generation of a variety of tumors [31, 32]. The two 4-node subnets (crosstalk and joint) have more common TFs, namely TP53 [33], SP1 [34], E2F4 [35], NFKB1 [36], and MYB [37]. They have been reported to be associated with lung cancer. However, they were not the hubs of triplet.

The subnets crosstalk and joint, which take co-expression and interaction into account, are annotated with the MAPK signaling pathway, the ErbB signaling pathway and the p53 signaling pathway. Most of the lung adenocarcinoma-related drugs, such as gefitinib [38] and tarceva [39], play a role by interrupting the signaling pathways. Therefore, the related genes, miRNAs and TFs could be targeted to inhibit tumor growth. Furthermore, the 10-gene biomarker extracted from joint was significantly enriched in the ErbB signaling pathway and the MAPK signaling pathway. These results indicate that the 10-gene biomarker was significantly linked to cancer and thus could be a potential drug target. The survival analysis of the biomarker also indicated its significant correlation with lung adenocarcinoma. After literature identification, seven of the ten genes were found to be associated with lung cancer, while the other three, *JUN*, *NR3C1*, and *GRB2*, have not been reported to be correlated with lung cancer. Among these, *JUN* is a known oncogene. Mathas et al. [40] found it was associated with lymphoma. *NR3C1* is a glucocorticoid receptor, and Lind et al. [41] confirmed it was epigenetically deregulated in colorectal tumorigenesis. *GRB2* can bind the epidermal growth factor receptor (EGFR), and Daly et al. [42]reported it to be associated with breast cancer. It was thus inferred that *JUN*, *NR3C1*, and *GRB2* were most likely to be new candidate lung adenocarcinoma-related genes.

**Fig 4:**
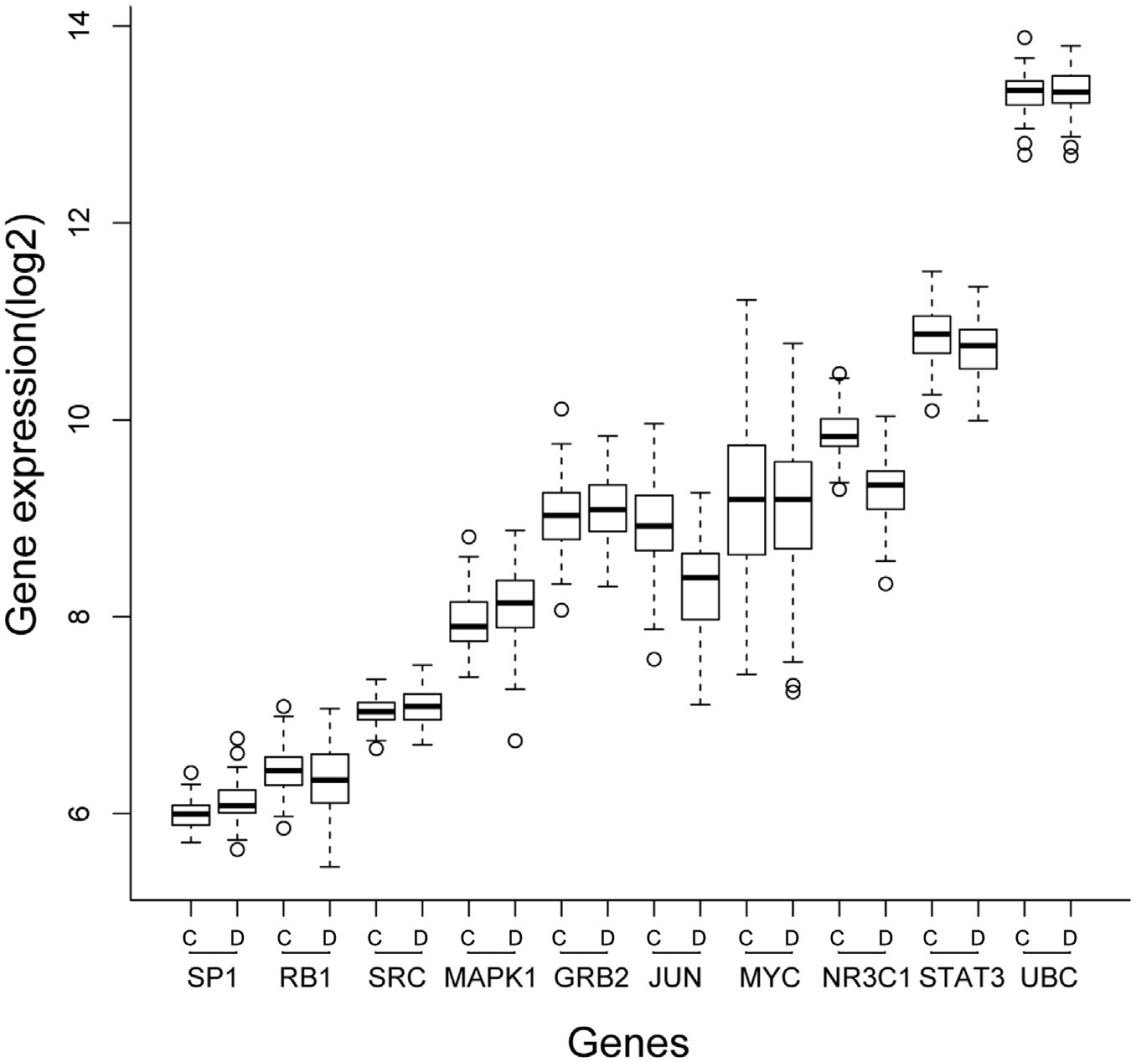
The boxplot of expressed comparison of the top ten genes in Joint. For each gene, the left box is the expression of control samples and the right box is the expression of disease samples. Only JUN and NR3C1 are differentially expressed between disease and control samples.

Among these ten genes, only *JUN* and *NR3C1* were identified as being differentially expressed genes (Fig 4). They are all lung adenocarcinoma-related genes specifically detected by differential interactions. This further verified the robustness of our approach. *JUN* and *NR3C1* were detected by both differential interactions and by differential expression, increasing their possibility to be correlated with lung adenocarcinoma.

Our method has been compared with other studies of TF and miRNA co-regulation. In the present paper, the construction of the regulatory network not only considered the case of feed-forward and feed-back loops of the three nodes, but also included 4-node loops which considered the co-expression and interaction between DIGs. In this case, we have made the results more comprehensive. All the genes we used in the network (DIGs) were predicted to associate with cancers, which should make the results even more persuasive. Our work could be complementary to the high-throughput experimental methods. This view was confirmed by the experiment of Sun et al [19]. Our method based on differential interactions considered the difference among cancer-related genes from a dynamic level viewpoint, which ensured the genes were more representative. Furthermore, the established analytical methods might also be used to study other complex diseases.

Since copy number variation, DNA methylation and mutation might affect gene expression, we look forward to joining other types of data to improve our work in the future.

## Materials and Methods

### Data

Three GEO (Gene Expression Omnibus)(http://www.ncbi.nlm.nih.gov/geo) lung adenocarcinoma expression profiles from the same platform (GPL570) were acquired: GSE31547 (30 primary lung adenocarcinomas and 20 adjacent normal lung controls), GSE10072 (DOI: 10.1371/journal.pone.0001651)[43] (58 tumor and 49 non-tumor tissues), and GSE7670 (DOI:10.1186/1471-2164-8-140)[44] (pairwise samples from 27 patients) (S5 Table). The profiles were processed separately. A probe was removed if it corresponded to more than one gene, and the values were averaged if multiple probes corresponded to the same gene. Finally, missing values (<5%) were filled by the K-means method, and the data were standardized. To eliminate the batch effect of different profiles, original expression values were replaced with a rank for each sample.

Human protein-protein interaction (PPI) data for the global protein-protein interaction network (PPIN) were obtained from nine databases: BioGRID, BIND, HPRD, IntAct, MINT, MIPS, PDZBase, DIP and Reactome. Redundant data and interactions that had not been confirmed by experiments or predicted in the literature were deleted [45].

### Detection of lung adenocarcinoma-related genes by differential interactions

In the present paper, we have developed a new method to identify cancer-related genes according to the interactional differences between cancer and normal samples, which we call differential interactions. These genes are likely to play important roles in the pathogenesis and progression of cancer, as they behave dissimilarly in cancer samples.

**Fig 5:**
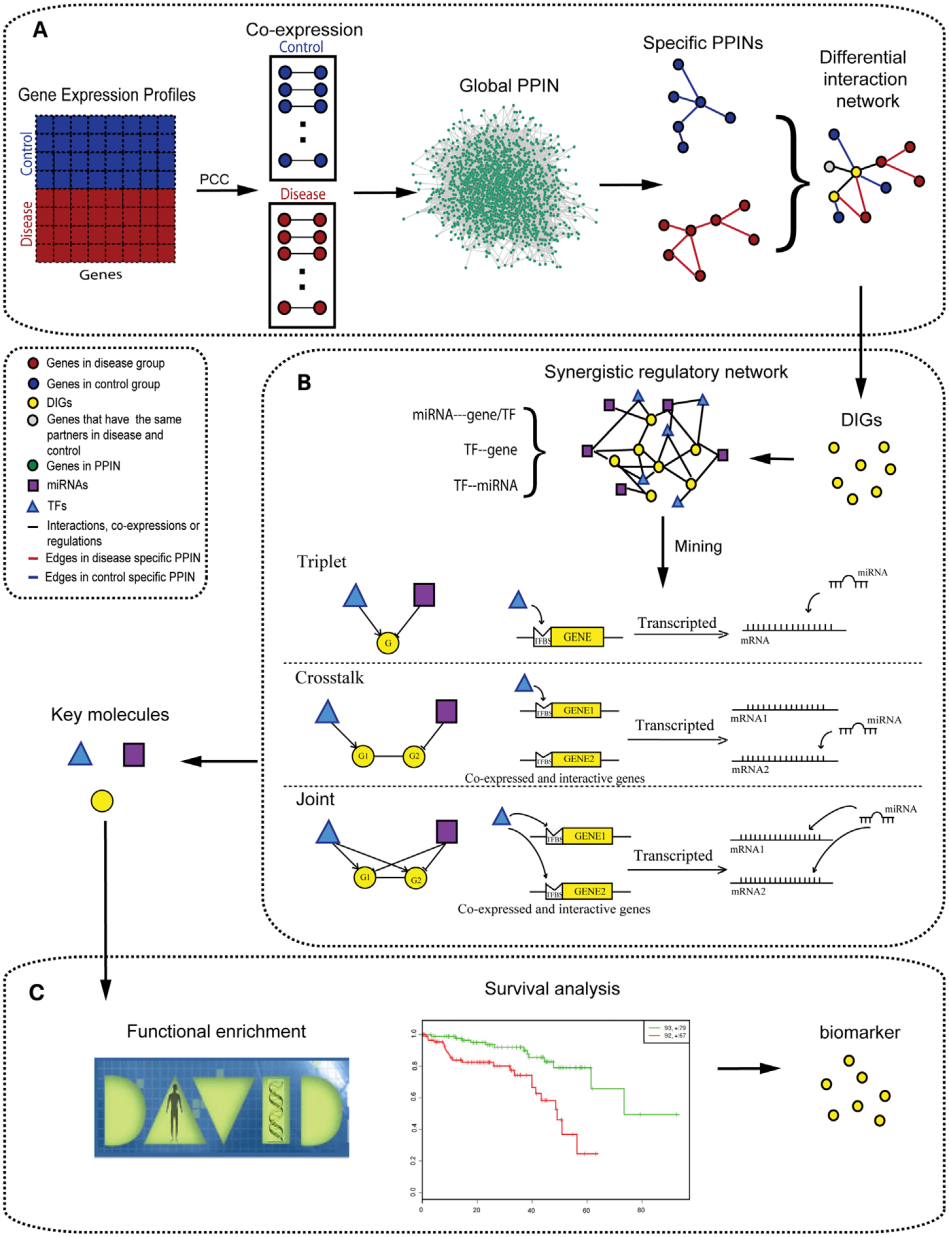
Flow diagram of this work. Detection of differential interaction genes. The gene expression profiles were separated into disease and control groups. The co-expressed gene pairs were then obtained from Pearson correlation coefficient (PCC). The gene pairs were mapped to global protein-protein interaction network (PPIN) to obtain specific PPINs. Finally, the two specific PPINs were merged to a differential interaction network (DIN) to detect DIGs. (B) Excavation of the three co-regulatory motifs. MiRNA&TF co-regulated relationship were introduced to our DIGs to construct a miRNA&TF synergistic regulatory network. Three kinds of motifs (triplet, crosstalk, and joint) were mined in the network. (C) Identification of biomarker. Key molecules were detected from the motifs. Functional enrichment and survival analysis were applied to gain biomarker.

The basic idea of lung adenocarcinoma-related gene detection is to obtain disease/control specific PPINs through the overlap of co-expression and interaction, and by predicting the lung adenocarcinoma-related genes through differences between interactions of disease and control samples. First, an expression profile was divided into disease and control samples. Then, co-expressed gene pairs were calculated according to the Pearson correlation coefficient (γ≥0.75 and p≤0.05) for the two groups, respectively. The p-value was computed by transforming the correlation to create a t statistic having n-2 degrees of freedom, where n is the number of rows of data. At the same time, a global PPIN was constructed based on the human PPI data. Subsequently, co-expressed genes were mapped to global PPIN to obtain specific PPINs for the disease and control groups, respectively. Finally, the two specific PPINs were compared to detect lung adenocarcinoma-related genes. The common genes (DIGs) of the two specific PPINs were extracted if they had different interaction partners in the two networks (Fig 5A). These DIGs have different interactions under normal and disease conditions. We assume that they are potential lung adenocarcinoma-related genes.

### Target prediction of miRNAs and TFs

MiRNA-target data were predicted by StarBase [46]. The following parameters were selected for reducing the false positives in the data during processing: (i) Number of supporting experiments > = 5, meaning that at least five CLIP-Seq experiments supported the predicted miRNA target site; (ii) Pan-Cancer > = 3, meaning that the expression of miRNA and the target gene was anti-correlation (Pearson correlation: γ<0, p-value<0.05) in at least three cancer types. The miRNA-TF regulations were extracted from the miRNA-target data.

TF-target data were predicted by four databases, ORegAnno [47], PAZAR [48], TRANSFAC [49], and TRED [50]. In order to obtain comprehensive regulatory information of the TFs and target genes, we combined the four databases.

The information about pre-miRNAs was obtained from miRbase [51]. In this paper, the region 2 kb upstream of pre-miRNAs was considered as the promoter region. Then, the conserved TFs binding sites were searched in this region using the UCSC genome browser (Z score = 2.33). Subsequently, the target relationships of miRNAs to TFs were collected from TransmiR [52] and manually curated from a large number of published articles [53].

### Co-regulatory motifs

Based on the predicted regulations, a synergistic regulatory network was constructed, following which, the co-regulatory motifs of miRNAs, TFs and genes were mined from the network (Fig 5B). We assumed that co-expressed and interacting genes tended to participate in cancer-related biological functions together. According to which DIGs were investigated, co-expression and interaction data revealed three types of motifs (triplet, crosstalk and joint) were defined. Triplet is a 3-node feedforward loop (FFL), which only considers the co-regulation of a pair of miRNA and TF to one gene. Crosstalk and joint are both 4-nodes with a pair of miRNA and TF and two DIGs. The two DIGs are co-expressed and interacted. In crosstalk, the TF regulates one of the two genes and the miRNA regulates the other. In joint, the TF/miRNA regulates both of the co-expressed and interacted genes simultaneously.

### Significance test

The hypergeometric test p-values were calculated for the obtained hub miRNAs.

$$P=1-\sum_{i=1}^{m}\frac{\left(\begin{array}{c} M\\ i \end{array}\right)\left(\begin{array}{c} N-M\\ n-i \end{array}\right)}{\left(\begin{array}{c} N\\ n \end{array}\right)}$$

Where, n represents the total number of target genes of the miRNA, N is the total number of coding genes in the human genome, M stands for the total number of lung adenocarcinoma-related genes (1,791), and m represents the number of lung adenocarcinoma-related genes that the miRNA targets.

To minimize the effect of false positives in the miRNA-target data, 1,000 randomization tests were conducted to ensure the biological significance of the detected miRNAs. In these, 1,791 genes were randomly selected from all the coding genes in the human genome and assessed as lung adenocarcinoma-related genes. The False Discovery Rate (FDR) threshold for the simulation p-values was set to 0.01.

To test the significance of the motifs recovered from the regulatory network, the network was tested with 1,000 times permutations under the circumstances of a constant degree distribution. Then, the three kinds of motifs, triplet, crosstalk, and joint, were searched in each random network, and their significant P values were calculated respectively:

$$P=\frac{N_{high}}{1000}$$

Where, *N*_*high*_ is the number of random networks with more motifs than in the real network. Then the Z-value was defined:

$$Z-value=\frac{N_{real}-N_{mean}}{SD}$$

Where, *N*_*real*_ represents the number of motifs in the real network, *N*_*mean*_ indicates the average number of motifs in 1000 random networks, and *SD* represents the standard deviation of 1000 random networks. The Z-value calculates the distance between the true value and the random mean by the unit of standard deviation. The difference between the true and random N is larger with an increasing Z-value.

### Functional analysis

Function enrichment analysis (KEGG pathway and Gene Ontology BP) of the co-regulated related genes of the cachets of each motif was carried using DAVID (http://david.abcc.ncifcrf.gov/). The significance threshold was set to FDR ≤ 0.01.

### Survival analysis

The top ten hub genes of joint were extracted as a biomarker. To verify whether the identified biomarker was associated with patient survival, the survival analysis was performed using the survival package in R, based on the prognostic index (*PI*) to generate the risk groups:

$$PI=\beta_{1}x_{1}+\beta_{2}x_{2}+\cdots +\beta_{p}x_{p}$$

Where, *p* represents for the number factors in the analysis, *x*_*p*_ is the expression of the *p*th gene and *β*_*p*_ is calculated through the COX regression. *PI* is an important factor in disease risk assessment. An increasing of *PI* suggests the survival time of the patients will gradually shorten. In this work, samples were ranked based on *PI*, and then the samples were separated into two equal size groups: a high risk group and a low risk group, based on the median of *PI*. The differences between the survival curves of the high and low risk groups showed whether the detected biomarker was significantly associated with prognosis.

## Supporting Information

S1 Table The 1,791 DIGs. (XLSX)

S2 Table The cachets of the three subnets. (XLSX)

S3 Table The KEGG&GO annotation of the three motifs. (XLSX)

S4 Table The KEGG&GO annotation of the ten hub genes in Joint. (XLSX)

S5 Table The samples of expression profiles. (XLSX)

S6 Table The compare of detected genes by differential interaction, differential expression and differential co-expression. The overlap with Cosmic gene set was marked with red.
(XLSX)

S1 Fig The heatmaps of samples hierarchical clustering by 1,791 DIGs. The heatmaps of (A) GSE31547 and (B) GSE7670.
(EPS)

S2 Fig The survival analysis result of top ten genes of Crosstalk. (EPS)

S3 Fig The survival analysis result of top five genes of Joint. The top five genes are UBC, SRC, SP1, MYC and STAT3.
(EPS)

S4 Fig The survival analysis result of top 20 genes of Joint. The top 20 genes are UBC, SRC, SP1, MYC, STAT3, JUN, NR3C1, RB1, GRB2, MAPK1, CDKN1A, MDM2, CREBBP, CCND1, EP300, RELA, FOS, UBE2I, PCNA and TGFBR2.
(EPS)
